# Development and characterisation of a decellularised bovine osteochondral biomaterial for cartilage repair

**DOI:** 10.1007/s10856-015-5517-0

**Published:** 2015-04-21

**Authors:** Hazel L. Fermor, Serena L. Russell, Sophie Williams, John Fisher, Eileen Ingham

**Affiliations:** Faculty of Biological Sciences, University of Leeds, Leeds, UK; School of Mechanical Engineering, University of Leeds, Leeds, UK

## Abstract

It is proposed that an acellular natural osteochondral scaffold will provide a successful repair material for the early intervention treatment of cartilage lesions, to prevent or slow the progression of cartilage deterioration to osteoarthritis. Here, we investigated the efficacy of methods for the decellularisation of bovine osteochondral plugs. The plugs were subject to four freeze/thaw cycles followed by two cycles of washes in hypotonic solution and low concentration (0.1 % w/v) sodium dodecyl sulphate with protease inhibitors. Plugs were treated with nuclease (DNase and RNase) treatment followed by sterilization in peracetic acid. Full tissue decellularisation was achieved as confirmed by histological analysis and DNA quantification, however the resultant acellular matrix had reduced glycosaminoglycan content which led to an increased percent deformation of cartilage. Furthermore, the acellular scaffold was not reproducibly biocompatible. Additional terminal washes were included in the process to improve biocompatibility, however, this led to visible structural damage to the cartilage. This damage was found to be minimised by reducing the cut edge to cartilage area ratio through decellularisation of larger cuts of osteochondral tissue.

## Introduction

Osteoarthritis (OA) is the progressive degeneration of natural joint tissues including articular cartilage, bone and supporting ligaments which results in pain and loss of motion for sufferers [1]. OA is the most common disorder affecting joints [2], in the UK an estimated 8.75 million people aged 45 and over sought treatment for the disease [3]. The causes of OA are multifactorial and not fully understood. One known cause of OA is the development of initial cartilage lesions, often as a result of joint trauma [4]. These lesions are unable to heal as the tissue is avascular, so progressively deteriorate over time with normal joint loading and activity [5].

Current surgical interventions to repair initial cartilage lesions, such as marrow stimulation techniques [6], autologous mosaicplasty [7], autologous chondrocyte implantation [8] and matrix-induced chondrocyte implantation [9] have been reported to have variable clinical outcomes. Many treatments do not produce a hyaline-like cartilage repair, leading to uncertain long term prognosis.

Due to the limitation of current interventions, the repair of cartilage lesions using tissue engineered approaches is being explored. Synthetic biomaterials such as polycaprolactone (PCL) [10] and polylactic acid [11] are easily manufactured with precise material properties; however achieving satisfactory biological integration is often a challenge. Biological materials such as fibrin [12] and gelatin [13] are biodegradable and biocompatible, however there remain concerns over scaffold integration.

Due to the complex biological and biochemical structure of natural articular cartilage the tissue exhibits extraordinary biomechanical and frictional properties [14]; this is difficult to recapitulate using conventional biomaterial approaches, although advances are being made. Recently, anisotropic cartilage biomaterials have been produced by electrospinning PCL fibres. This nanofabrication technology enabled tangential alignment of fibres at the surface and random orientation in the rest of the material, increased fibre diameter was included in the base of the material to mimic natural cartilage zonal microstructure showing promising in vitro results [10].

An alternative approach is decellularisation of natural tissues. Decellularisation of natural tissues has been shown to produce extracellular matrix (ECM) scaffolds with the same structure and function as the original tissues whilst removing immunogenic cells [15, 16]. This approach has led to the clinical translation of acellular allogeneic and xenogeneic tissues for use in cardiovascular [17] and connective tissue [18, 19] applications. This approach has also been investigated to develop acellular cartilage and osteochondral scaffolds for use in cartilage lesion repair [20–23]. It is proposed that an acellular osteochondral scaffold will have superior biological and biomechanical characteristics and will show improved integration compared to other tissue engineered cartilage repair materials, due to the presence of the subchondral bone. Kheir et al. [21] presented initial data on the decellularisation of porcine osteochondral tissues from 4–6 month old pigs using low concentration sodium dodecyl sulphate (SDS) and protease inhibitors [15, 16]. However, mature bovine tissue may be a more appropriate source material [24]. Here, we present data on the development of an improved method to decellularise mature bovine osteochondral plugs (clinically relevant in mosaicplasty-like procedures). The resultant acellular scaffold was analysed using biological, biochemical and biomechanical methods and the biocompatibility of the material was determined.

## Materials and methods

### Tissue preparation and decellularisation of osteochondral plugs

Osteochondral plugs (9 mm diameter, 12 mm thickness) were extracted from the medial patello-femoral groove of 18 month old bovine knee joints using bespoke corers and a hand held electric drill. The osteochondral plugs were either retained as native (n = 5 from 5 cows) or decellularised using one of five iterative methods based on the processes described by Booth et al. [15], Stapleton et al. [16] and Kheir et al. [21].

#### Process (1)

Osteochondral plugs (n = 5 from five cows) were frozen at −20 °C followed by thawing at 42 °C, this process was repeated once more then a further two times with the plugs submerged in a hypotonic solution (10 mM tris–HCl, pH 8.0; Sigma plus 10 KIU ml^−1^ aprotinin; Nordic Pharma). Thawed plugs were washed for 3 × 10 min in phosphate buffered saline (PBS; Oxoid) and washed in hypotonic solution for 18 h, followed by 24 h in hypotonic solution containing SDS (0.1 % (w/v); Sigma). Plugs were then washed 3 × 10 min in PBS before being incubated in nuclease solution (50 mM Tris solution, pH 7.5, with 10 mM magnesium chloride, 50 U ml^−1^ DNAase and 1 U ml^−1^ RNAase; Sigma). Following a further 3 × 10 min washes in PBS plugs were sterilised using peracetic acid (PAA; 0.1 % v/v in PBS; Sigma). Finally plugs were washed 3 × 10 min in PBS and stored at −20 °C until analysed. All incubations were carried out at 42 °C with the exception of the nuclease treatment which was performed at 37 °C and PAA sterilisation which was performed at 27 °C. All washes were performed with 125 ml solution with agitation on an orbital shaker. The washes following PAA sterilisation were performed aseptically in a Class II safety cabinet.

#### Process (2)

This method differed from process (1) only in that there was an extended terminal 36 h wash in PBS added to the end of the process (n = 5 plugs from three cows).

#### Process (3)

This method was the same as process (2) with the addition of use of a dental water flosser (Water Pik, Inc.) to physically remove the bone marrow from the osteochondral plugs with a fine jet of PBS (400 ml per plug). This was performed following the first freeze/thaw cycle (n = 5 plugs from three cows).

#### Process (4)

This method was the same as process (3) with the addition of an incubation of the plugs PBS at 42 °C for 18 h following the first freeze/thaw cycle prior to the use of the water flosser (n = 5 plugs from three cows).

#### Process (5)

This method was the same as process (4) with an additional 24 h wash in hypotonic solution and 24 h wash in SDS in hypotonic solution after the original SDS wash (n = 5 plugs from three cows).

#### Extended washes

PBS washes (4 × 24 h) were added at the end of process (5) using n = 5 plugs from three cows.

### Histological analysis

Osteochondral plugs (n = 5 native, n = 5 decellularised using process 1; n = 3 decellularised using processes 2–5) were fixed for 48 h in 10 % (v/v) neutral buffered formalin (NBF; Bios Europe Ltd), before being decalcified in 12.5 % (w/v) EDTA (pH 7: Fisher Scientific) for 4 weeks or until soft enough to be cut with a scalpel. Plugs were bisected before being dehydrated and paraffin wax and embedded using an automated process (Lecia TP 1020, Lecia Microsystems). Sections of 6 μm thickness were cut through the cartilage surface into the bone, encompassing the different cartilage zones and subchondral bone. Haematoxylin and eosin (H&E; Bios Europe Ltd) staining was used to assess tissue histoarchitecture. DAPI (Sigma) staining was used to visualise cell nuclei. Safranin O and Fast Green (Sigma) staining was used to visualise glycosaminoglycan (GAG) distribution.

### Biochemical analysis

#### DNA quantification

The Qiagen DNeasy blood and tissue kit was used to digest cartilage and extract and purify tissue DNA according to the manufacturers’ instructions (Qiagen). Cartilage (n = 5 native, n = 5 decellularised using process 1; n = 3 decellularised using processes 2–5) was cut away from the subchondral bone and digested using proteinase K (Qiagen). Purified DNA was quantified using a nanodrop nano spectrophotometer (Nanodrop). DNA content is expressed in ng mg^−1^ of wet weight of tissue.

#### Sulphated sugar quantification

Lyophilised cartilage (n = 5 native, n = 5 decellularised using process 1; n = 3 decellularised using processes 2–5) was digested using papain solution (5 ml, 50 U ml^−1^ papain, Sigma, in PBS at pH 6 with 5 mM l-cysteine hydrochloride, Sigma, and 5 mM EDTA) for 48 h at 60 °C. GAG content was quantified following a method adapted from Farndale et al. [25]. Briefly, standard concentrations of chondroitin B sulphate (Sigma) were produced, 1,9-dimethylmethylene blue (Sigma) solution (250 μl) was added to each standard and sample (40 μl) in a clear flat bottomed 96-well plate, the plate was agitated for 2 min before the absorbance was read on a spectrophotometer (Multiscan Spectrum, Thermo Lab Systems) at 525 nm. Interpolation of sample absorbance from the standard curve gave the GAG concentration of each digest which was then expressed in µg mg^−1^ of dry weight of tissue.

#### Collagen quantification

Lyophilised cartilage (n = 5 native, n = 5 decellularised using process 1; n = 3 decellularised using processes 2–5) was hydrolysed in 6 M hydrochloric acid (HCl, VWR) for 16 h at 80 °C followed by neutralisation with 6 M sodium hydroxide (NaOH: Fisher). Hydroxyproline content was quantified following a method adapted from Edwards & O’Brien [26]. Briefly, standard concentrations of trans-4-hydroxy-l-proline (Sigma) were produced, Chloramine-T oxidation solution (100 μl; Sigma) was added to each standard and sample (50 μl) in a clear flat bottomed 96-well plate, the plate was agitated for 5 min. Ehrlich’s reagent (100 μl; Sigma) was then added and the plate was incubated in a water bath at 60 °C for 45 min before the absorbance was read at 570 nm on a spectrophotometer. Interpolation of sample absorbance from the standard curve gave the hydroxyproline concentration of each sample. This was then expressed as µg mg^−1^ dry weight of tissue.

### Compressive testing using an indenter

The methods used have been previously described by Pawaskar et al. [27] and Taylor et al. [28] to assess the biomechanical properties of cartilage attached to bone. Briefly, osteochondral plugs (n = 5 native, n = 5 decellularised using process 1; n = 3 decellularised using processes 2–5) were compressed in a purpose built indentation rig using a 3 mm diameter, hemispherical, stainless steel indenter under a load of 0.8 N. Plugs were submerged in PBS during testing to maintain cartilage hydration. The deformation of cartilage was measured at a sampling frequency of 5 Hz over 1 h, after which all samples had reached equilibrium. Following compression, plugs were fully rehydrated in PBS before cartilage thickness was measured. A needle indenter was used to penetrate the cartilage, lowering at a rate of 4.5 mm min^−1^; the resistance to motion was measured using a 500 N load cell (Instron 3365). An increase in load was recorded when the needle first contacted the cartilage surface and a second increase when entering the bone, the distance between these two changes in load was taken as the cartilage thickness. Deformation of cartilage was normalised to thickness to give percentage deformation for each osteochondral plug.

### Biocompatibility testing

#### Contact cytotoxicity

Baby hamster kidney cells (BHK, Health Protection, England) were cultured in BHK culture medium (GMEM; Sigma, 5 % (v/v) foetal bovine serum (FBS), SeraLab, 10 % (v/v) tryptone phosphate broth, 2 mM l-glutamine, 100 U ml^−1^ penicillin and 100 mg ml^−1^ streptomycin, Sigma) at 37 °C in 5 % (v/v) CO_2_ in air. 3T3 murine fibroblasts (European Collection of Cell Cultures) were cultured in 3T3 culture medium (DMEM; Sigma, 10 % (v/v) FBS, 2 mM l-glutamine, 100 U ml^−1^ penicillin and 100 mg ml^−1^ streptomycin) at 37 °C in 5 % (v/v) CO_2_ in air. Decellularised osteochondral tissue samples (n = 3, 1 mm × 3 mm × 5 mm) were attached to the centre of 6-well culture plates using 3 M Steri-strips (Medisave). Steri-strips and cyanoacrylate were applied to separate wells in triplicate to act as negative and positive controls respectively. BHK or 3T3 cells were seeded into each well at the appropriate cell density to achieve confluence under usual culture conditions after 48 h. Following culture with tissue samples and controls for 48 h at 37 °C in 5 % (v/v) CO_2_ in air cells were examined using phase contrast microscopy to visualise changes in cell morphology and confluence.

#### SDS quantification

In order to determine the levels of SDS remaining in the osteochondral tissues following processing, decellularisation (process 5) was performed using SDS in hypotonic solution which had been spiked with ^14^C radiolabelled SDS (0.001 µCi ml^−1^; Hartmann Analytical). Macerated decellularised cartilage or bone (40 mg) were added in triplicate to a flat bottomed 96 well OptiPlate™ (Perkin Elmer) and MicroScint™-20 (160 µl; Perkin Elmer) was added to each well. Counts per minute were measured using Top count™ (Perkin Elmer), with each well being counted for 20 min. A standard curve of counts per minute was produced from known concentrations of SDS, from which the concentration of SDS in the test tissue could be interpolated.

#### SDS cytotoxicity

To determine the concentration at which SDS exhibited toxic effects upon mammalian cells, BHK and 3T3 cells were cultured in triplicate in 96-well culture plates for 24 h; culture medium was aspirated and replaced with BHK or 3T3 medium containing a range of known concentrations of SDS. BHK or 3T3 culture medium and 40 % (v/v) dimethyl sulphoxide (DMSO; Sigma) were used as negative and positive controls for cell toxicity respectively. Cells were cultured for a further 24 h at 37 °C in 5 % (v/v) CO_2_ in air and the ATP content measured using the ATPLite-M^®^ assay kit (Perkin Elmer) according to the manufacturers’ instructions to quantify cell viability.

### Statistical analysis

Numerical data was analysed using Microsoft Excel (version 2010, Microsoft) and is presented as the mean (n = 5 or n = 3) ±95 % confidence levels (CL). One way analysis of variance (ANOVA) was performed and individual differences between group means were identified by calculating the minimum significant difference at *P* = 0.05 using the T-method, or T’-method when comparing groups of unequal sample size [29].

## Results

**Fig. 1:**
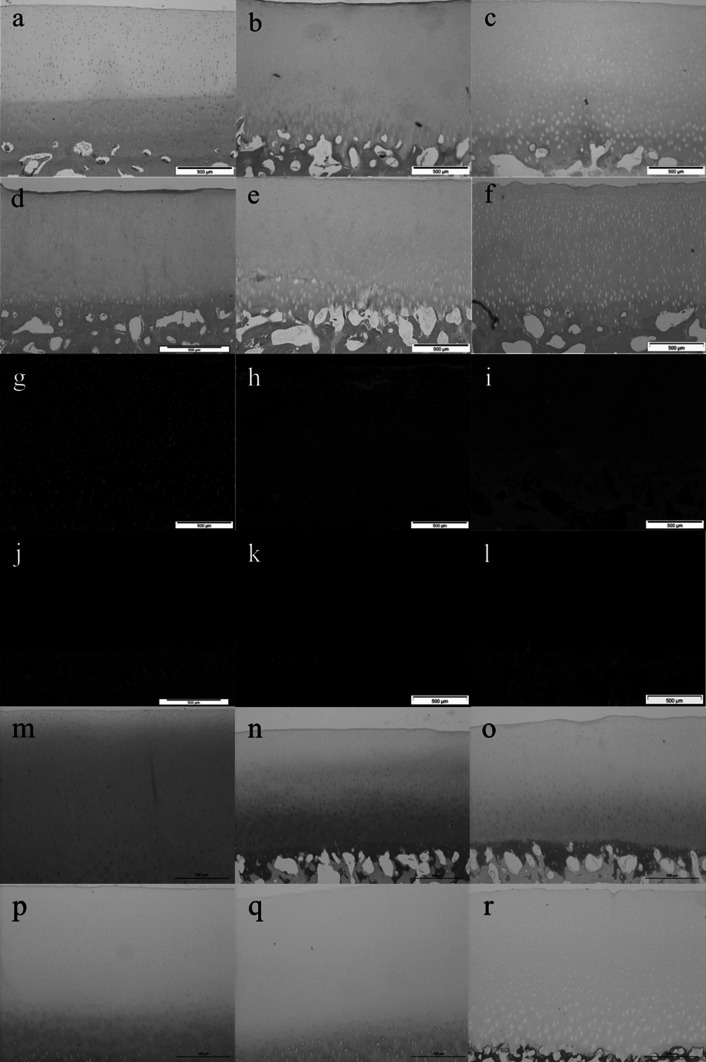
H&E, DAPI and Safranin O stained sections of native and decellularised bovine osteochondral plugs. H&E: **a** native, **b** decellularised using process (1), **c** process (2), **d** process (3), **e** process (4), **f** process (5). DAPI: **g** native, **h** decellularised using process (1), **i** Process (2), **j** Process (3), **k** process (4), **l** process (5). Safranin O: **m** native, **n** decellularised using process (1), **o** process (2), **p** process (3), **q** process (4), **r** process (5).* Scale bar* = 500 µm

**Fig. 2:**
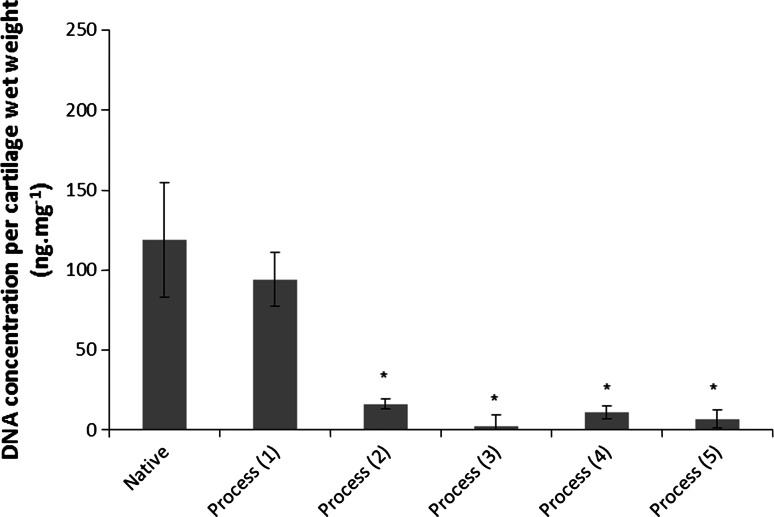
Total DNA content of native and decellularised cartilage. Data is shown as the mean (native and decellularised by process (1) n = 5, others n = 3) ±95 % confidence limits. * indicates significant difference compared to native tissue *P* < 0.05 (ANOVA)

**Fig. 3:**
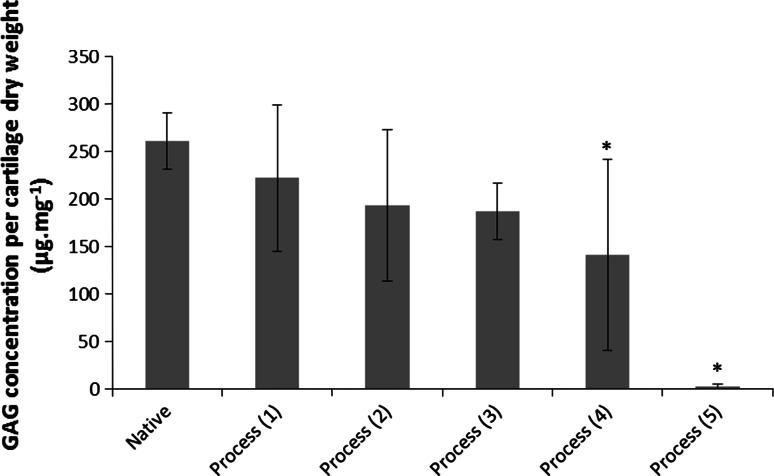
GAG content of native and decellularised bovine cartilage. Data is shown as the mean (native and decellularised by process (1) n = 5, others n = 3) ±95 % confidence limits. *indicates significant difference compared to native tissue *P* < 0.05 (ANOVA)

**Fig. 4:**
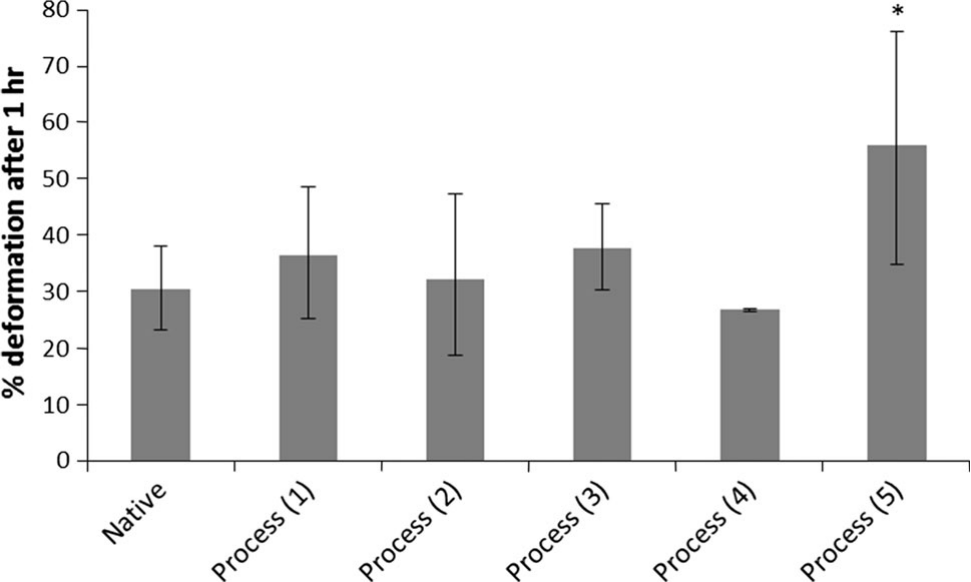
Percent deformation of native and decellularised cartilage. Data was subject to arcsine transformation prior to calculation of the 95 % confidence limits and analysis of variance. Data is shown as the back transformed mean (native and decellularised using process (1) n = 5, others n = 3) ±95 % confidence limits. * indicates significantly different compared to native tissue *P* < 0.05 (ANOVA)

Initial attempts to decellularise bovine osteochondral tissues using process (1) were not successful and this lead to numerous iterations of the decellularisation process which are documented below. Images of sections of native bovine osteochondral tissue and tissue following the various decellularisation processes stained with H&E, DAPI and Safranin O are presented in Fig. 1. The total DNA and GAG content of native bovine cartilage and the cartilage following the various decellularisation processes are presented in Figs. 2 and 3. The data for the percentage deformation of the native bovine osteochondral tissues and tissues following the various decellularisation processes are presented in Fig. 4.

### Process (1)

Following application of decellularisation process (1) staining of treated tissue sections with H&E and DAPI revealed that there were still a number of cells present in the cartilage and bone (Fig. 1b, h). The general structure of the tissue was maintained, however marrow was still present in the bone. There was no significant reduction in the total DNA content of the cartilage compared to native tissue (Fig. 2). The GAG content (222.1 ± 77.1 µg mg^−1^) of cartilage following decellularisation process (1) was not significantly different from native tissue (261.1 ± 33.3 µg mg^−1^; Fig. 3); however a reduction was observed in sections of the superficial and middle zones compared to native cartilage when stained with Safranin O (Fig. 1n). The percentage deformation (37.5 + 12.0/−11.2 %) of cartilage decellularised using process (1) was not significantly different from native tissues (30.4 + 7.6/−7.1 %; Fig. 4).

### Process (2)

Following the introduction of an extended PBS wash at the end of decellularisation Process (1), sections of osteochondral plugs decellularised using Process (2) showed a much greater reduction in cell content. Nucleated cells were, however, still visible in the deep/calcified cartilage regions and in the subchondral bone (Fig. 1c, i). Total DNA content (16.2 ± 3.1 ng mg^−1^) showed a significant reduction compared to native bovine tissue (119.0 ± 35.9 ng mg^−1^; Fig. 2). There was no significant difference in the GAG content of the treated cartilage (193.4 ± 79.9) compared to native cartilage (Fig. 3), however histologically a loss of GAGs throughout the cartilage tissue was seen (Fig. 1o). No significant difference was observed in the percentage deformation (32.2 + 15.2/−13.5 %) of the cartilage compared to native tissue (Fig. 4).

### Process (3)

To increase decellularisation solution access to the bone and deep cartilage layers a water flosser was used to remove the bone marrow from within the trabecular spaces. Visibly, little marrow was removed following application of the water jet. Histologically, no damage to the cartilage or bone was seen, however cells were still visible in the tissue within denser areas of bone (Fig. 1d, j). The total DNA content of cartilage (2.2 ± 7.4 ng mg^−1^) was significantly reduced compared to native cartilage (Fig. 2). There was no significant difference in the quantified GAG content of the treated cartilage compared to native cartilage (Fig. 3) or tissue biomechanics (Fig. 4), however histological analysis showed reduced GAG content throughout the tissue compared to native cartilage (Fig. 1p).

### Process (4)

The bovine osteochondral plugs were agitated in PBS for 18 h at 42 °C before treatment with the water flosser; this loosened the bone marrow and greatly improved ease and extent of bone marrow removal. A few cell nuclei however remained in the tissue at the cartilage-bone interface following treatment using process (4) Fig. 1e, k). The amount of DNA remaining in the cartilage (11.0 ± 3.9 ng mg^−1^) was again significantly reduced (Fig. 2). There was, however, a significant reduction in cartilage GAG content (141.0 ± 100.6 µg mg^−1^) compared to native tissue (Fig. 3) which was also seen histologically (Fig. 1q), although this did not have any effect on the percentage deformation (26.8 ± 0.3 %) of the tissue (Fig. 4).

### Process (5)

Application of process (5) to the bovine osteochondral plugs showed that two cycles of treatment with hypotonic solution and SDS were required to successfully remove all histological evidence of cell nuclei from the osteochondral tissues (Fig. 1f, l). DNA content per wet weight of tissue (7.0 ± 5.9 ng mg^−1^) was significantly reduced compared to native tissue (Fig. 2). The DNA content of cartilage decellularised using process (5) was 39.7 ± 34.1 ng mg^−1^ per cartilage dry weight. Quantification of the GAG content however revealed that almost all of the GAGs had been removed from the cartilage following decellularisation using process (5) (Fig. 3) with no Safranin O staining observed in the processed cartilage (Fig. 1r). This had an effect on the tissue biomechanics and the percentage deformation was increased to 55.9 % (+20.1/−21.2 %) compared to native tissue (Fig. 4). Cartilage decellularised using process (5) also showed a significant increase in hydroxyproline content (146.3 ± 65.1 μg mg^−1^) compared to native bovine cartilage (72.3 ± 26.5 μg mg^−1^) which had not been evident for cartilage decellularised using processes (1–4).

**Fig. 5:**
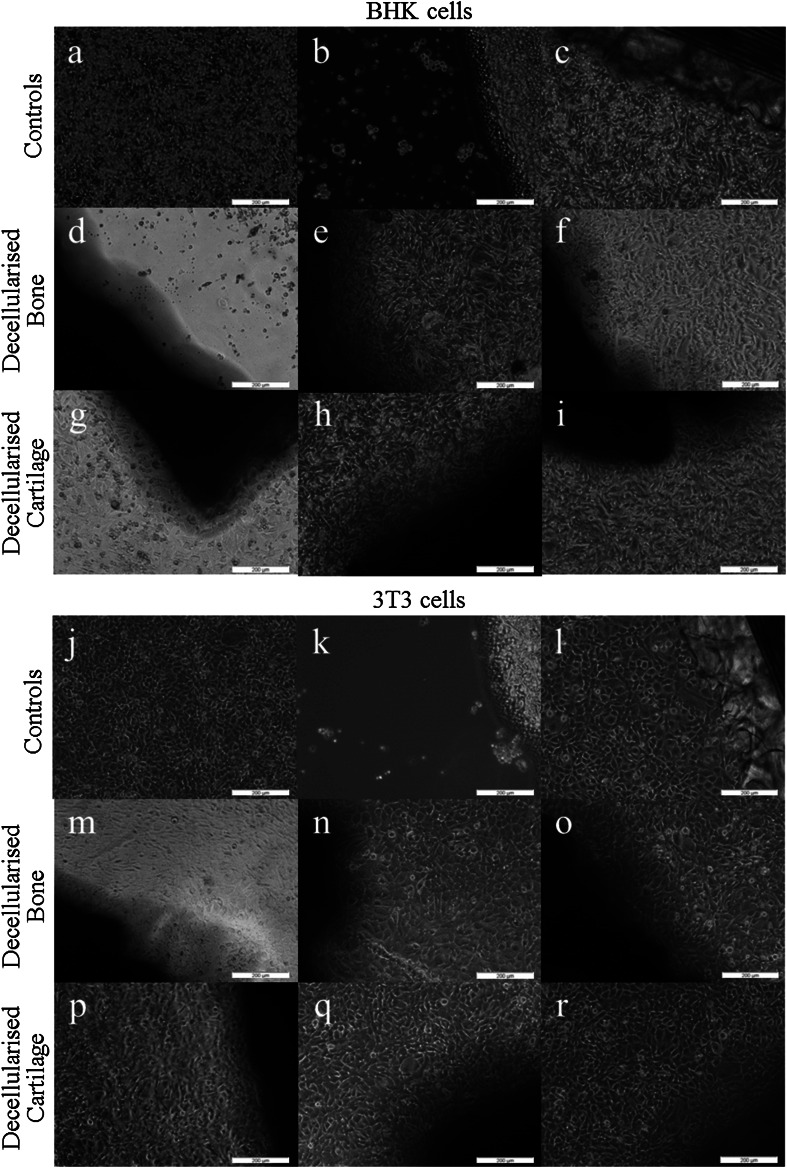
Contact cytotoxicity of decellularised bovine osteochondral tissues when cultured for 48 h with BHK and 3T3 cells. BHK cells: **a** cells only, **b** cyanoacrylate positive control, **c** steri-strip negative control, **d–f** decellularised bovine bone (n = 3), **g–i** decellularised bovine cartilage (n = 3). 3T3 cells: **j** cells only, **k** cyanoacrylate positive control, **l** steri-strip negative control, **m–o** decellularised bovine bone (n = 3), **p–r** decellularised bovine cartilage (n = 3)

Since the decellurisation process (5) appeared to be successful, it was necessary to determine whether the acellular tissue was biocompatible with cells. Culture of tissues (n = 3) decellularised using process (5) showed that all three samples of decellularised cartilage were compatible with BHK and 3T3 cells. Samples of decellularised bone (n = 3) were also compatible with 3T3 cells, however 1/3 samples of decellularised bone was cytotoxic to BHK cells (Fig. 5), with a zone of inhibited cell growth observable around the tissue.

Hence, the concentration of residual SDS in decellularised tissue was determined and this was found to be 364 ± 43 ng mg^−1^ per cartilage wet weight and 374 ± 187 ng mg^−1^ in bone. It was therefore relevant to determine the toxicity of varying levels of SDS to the two cell types. Culture of BHK cells with SDS showed a significant decrease in cell viability at SDS concentrations of 50 µg ml^−1^ and above whereas 3T3 cells only showed significantly reduced viability in the presence of SDS concentrations above 250 µg ml^−1^.

### Extended washes

**Fig. 6:**
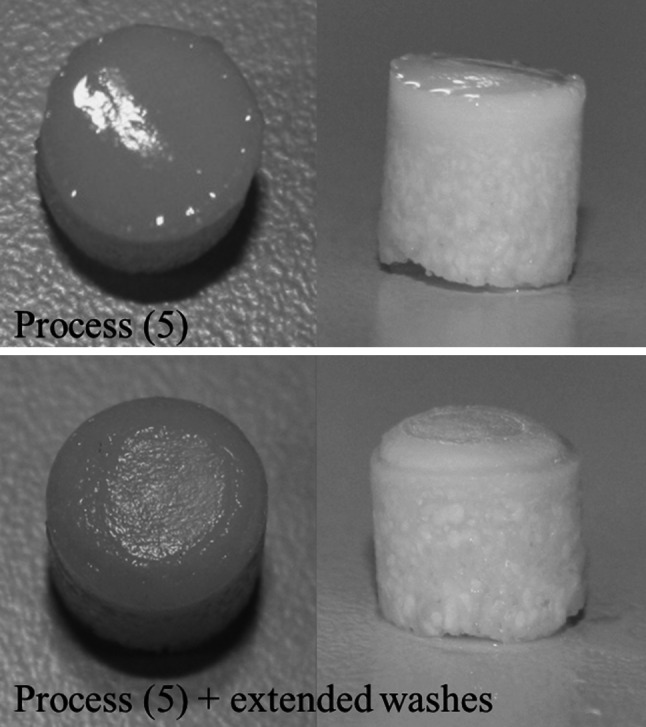
Macroscopic observation of osteochondral plugs decellularised using process (5) and process (5) with extended washes. *Top* panel shows smooth shiny cartilage following decellularisation with process (5). *Bottom* panel shows the dull, mottled cartilage surface (left) and the cartilage contracted in from the bone following decellularisation with process (5) with extended PBS washes

To improve biocompatibility by removing residual SDS a further 4 days of PBS washes were included at the end of decellularisation process (5). The addition of the extra wash step had a detrimental effect on the cartilage ECM, which became visibly damaged between the 2 and 3 day time point (Fig. 6), shrinking away from the bone or becoming completely detached. The remaining cartilage was contracted and softened having a gelatinous appearance and a roughened, dull surface.

## Discussion

The aims of this study were to investigate the utility of tissue decellularisation processes for the generation of acellular bovine osteochondral plugs of potential use in osteochondral lesion repair. The objectives were to develop a process which removed whole cells and nuclear material from the ECM scaffold to eliminate any adverse immunogenic effects of xenogeneic cellular antigens. Since the major structural macromolecules of the ECM, such as collagens, are relatively conserved throughout higher mammalian species, these matrix proteins should not evoke an immune response [30].

A protocol for decellularisation was developed, based on previous work by Booth et al. [19] and developments of the original protocol for porcine meniscus and osteochondral tissues [16, 21] which had shown that increased incubation temperatures were applied to overcome issues of the dense matrix and high GAG concentrations of cartilaginous tissues to improve the diffusion of decellularisation solutions. In a further change to the original process, EDTA which was used in the decellularisation process for soft tissues [19] as a metalloproteinase inhibitor was removed from the process to avoid decalcification of the bony component of the osteochondral plugs.

In the final iteration of the decellularisation process described here, histological analysis of bovine osteochondral plugs subject to process (5) showed removal of whole cells and large cellular debris, and the total DNA content of the cartilage was significantly reduced to 39 ng mg^−1^ per cartilage dry weight, below the recommended levels of 50 ng mg^−1^ double stranded DNA for tissue acellularity as established by Crapo et al. [31]. To achieve this level of decellularisation of the mature bovine osteochondral plugs, the various iterations of the process (processes (1–4)) showed that it was necessary to initially loosen and remove the bone marrow prior to subjecting the tissue to decellularisation solutions. This was achieved by incubation in PBS at 42 °C with agitation for 18 h and then use of a water flosser. It is therorised that removal of the bone marrow enabled improved diffusion of decellularisation solutions through the bone and up to the subchondral plate. Four cycles of freeze/thaw were required (two in hypotonic solution) to open up the ECM through formation of ice crystals to improve diffusion of solutions through the tissue. Two cycles of hypotonic buffer and low concentration SDS washes in series were required to remove the cells from the calcified cartilage and the subchondral bone plate, as these areas were most dense. This number of SDS cycles was fewer than required in the protocol developed by Khier et al. [21] for porcine osteochondral tissues which may have been due to the fact that the process used by Kheir et al. did not address removal of bone marrow or the smaller volume of tissue being decellularised, or indeed both. Extensive washing in PBS following nuclease treatment and PAA sterilisation was required to remove nucleic acids from the tissue (process 2 compared to process 3).

Although decellularisation was achieved, there were detrimental effects on the ECM. An almost complete loss of GAGs was observed in the cartilage decellularised using process (5) which had an adverse effect on the mechanical properties of the tissue resulting in a significant increase in cartilage deformation from 30 % in native tissue to 56 % in the decellularised tissue. Previous studies have found that decellularisation using SDS has led to removal of GAGs from tissue ECM [15, 16, 21]. Ionic interruption of link protein by SDS leading to disaggregation of aggrecan and hyaluronan, thus increased GAG mobility, has been suggested as a potential mechanism of GAG loss from tissues [21].

The DNA content of the bony section of the osteochondral tissue was not quantified; hence the residual DNA content of the bone is currently unknown. However, there was no evidence of cellular material in the bone as judged by histology and DAPI staining revealed no evidence of cell nuclei. Retention of some DNA fragments in the bone was not thought to be problematic since DNA alone will not elicit an immune response [32]. Residual DNA is known to result in calcification of decellularised tissues following implantation [33], however this would not pose an issue in mineralised tissue such as bone.

For acellular scaffolds to be used in patients, it is essential that they are not toxic or harmful. SDS, a detergent included in the decellularisation process to solubilise cellular membranes has been shown to have cytotoxic effects when not thoroughly removed from decellularised tissues [34]. SDS is able to impart a negative charge on proteins, which may inhibit cell attachment and proliferation which may affect cell survival [35]. Quantification of the residual SDS in the decellularised tissues clearly showed that the majority of the SDS was removed during wash steps, and that the decellularised bone and cartilage contained <37.5 ng mg^−1^ SDS per wet weight (circa 0.0375 % (w/w)), following decellularisation using process (5). The in vitro contact cytotoxicity tests carried out on these decellularised tissues showed inconclusive results. The acellular cartilage was biocompatible with both BHK and 3T3 cells. The acellular bone was biocompatible with 3T3 cells, however 1/3 acellular bone samples showed cytotoxicity with BHK cells. Further investigation revealed that the 3T3 cells were less sensitive to the cytotoxic effects of SDS in culture (sensitive to 250 μg ml^−1^ or 0.025 % (w/v)), than BHK cells (sensitive to 50 μg ml^-1^ or 0.005 % (w/v)), perhaps explaining the difference in cellular response to the acellular bone. It is likely that due to binding of the SDS to the 3D structure of the tissue, not all of the residual SDS in the tissue was available to interact with the cells in culture, thus although the levels of SDS remaining in both the cartilage and bone tissues were toxic the effects on the cells in culture were minimal.

Nevertheless, since the residual levels of SDS in the tissues was relatively high, further modifications to process (5) were investigated which involved increasing the number and duration of PBS washes at the end of the process to improve SDS wash out. When additional PBS washes were included, tissue samples began to show signs of damage. The cartilage contracted in from the bone and in some cases became detached. Schwarz et al. [22] produced an acellular scaffold from nasal cartilage. It was of interest to note, that although not recognised by the authors as damaged, the histology images of the decellularised cartilage showed similar morphology to the damaged cartilage found in the present study, they also identified a large reduction in GAG content.

Although the overall objective of this study, which was to develop a protocol for the successful decellularisation of bovine osteochondral plugs, was not achieved, this study has led to important knowledge regarding the difficulties of producing acellular osteochondral tissues. It is hypothesised that this is due to the unique structure of the cartilage ECM. The fibres of the collagen II network in cartilage have a characteristic orientation [36] and are under tension, providing the tensile strength of the tissue. Proteoglycans are dispersed throughout the collagen network and bind water in the tissue, providing the compressive strength of the tissue [37]. Preparing osteochondral tissues for decellularisation by cutting plugs would have severed the collagen fibres and resulted in loss of tension, particularly the fibres orientated parallel to the surface in the cartilage superficial zone. With the loss of tension, the collagen network would have had a reduced ability to counter the repulsive forces of negatively charged proteoglycans being held in close proximity to one another, resulting in an increase in porosity allowing proteoglycans to be washed out (GAGs removed), water to enter the tissue and the collagen fibres to therefore clump together in the remaining ECM.

In order to test the hypothesis that decellularisation of cuts of bovine osteochondral tissues (with a reduced ratio of cut edges to cartilage volume) would result in a tissue with less damage, a preliminary study was undertaken where by bovine osteochondral cuts of 20 mm × 40 mm × 12 mm thickness (n = 3) from the femoral groove were decellularised using process (5) plus 11 × 24 h extended PBS washes. Following decellularisation the cartilage did not detach from the bone or appear damaged macroscopically or microscopically, with the exception of the cut edges. It was therefore demonstrated that reducing the ratio of cut edges to cartilage area greatly improved cartilage stability during the decellularisation processes. However, the tissue was not completely decellularised. Research will now focus on developing a process for complete decellularisation of bovine osteochondral cuts, utilising the knowledge gained from this study, with a view to harvesting acellular osteochondral pins of clinically relevant size from the undamaged central regions of these decellularised large osteochondral constructs.

Decellularisation of cartilage for therapeutic applications has been approached in a number of ways. One approach is the homogenisation of cartilage tissue following lyophilisation and grinding [38] or shattering into fragments and centrifuging to produce microfilaments [39] prior to decellularisation. This produced a material that provided bioactive cues for cell growth, however lacked any 3D architecture essential for tissue biomechanical function. Decellularisation of intact 3D cartilage tissues has been investigated [20, 22] however both resultant scaffolds had inferior biochemical composition and thus biomechanical properties. Furthermore, the lack of subchondral bone in these cartilage scaffolds would limit the integration of implanted grafts. The decellularisation of immature porcine osteochondral tissues has been demonstrated [21], however the resultant scaffold again had reduced GAG content and therefore inferior mechanical properties. The current study investigated the decellularisation of a mature bovine osteochondral tissue. The method presented here is currently the most effective known for decellularisation of intact bovine osteochondral tissues, with no visible cell nuclei present and a residual DNA content of <50 ng mg^−1^ in the decellularised cartilage matrix. This protocol included the novel use of a water flossing technique to remove bone marrow, which enabled more complete decellularisation. Fewer cycles of SDS were required to fully remove cells compared to previously reported methodologies [21]. However, loss of GAG and reduced biomechanical properties was observed, as reported for other protocols [20–23].

## Conclusion

This study has increased knowledge and understanding of the effects of decellularisation processes on osteochondral tissues which will form the basis for future development of a bioactive, acellular, natural tissue engineered repair material for osteochondral lesions to prevent or delay the onset of OA.
